# A refined, rapid and reproducible high resolution melt (HRM)-based method suitable for quantification of global LINE-1 repetitive element methylation

**DOI:** 10.1186/1756-0500-4-565

**Published:** 2011-12-28

**Authors:** M Yat Tse, Janet E Ashbury, Nora Zwingerman, Will D King, Sherry AM Taylor, Stephen C Pang

**Affiliations:** 1 Department of Anatomy and Cell Biology, Queen's University, Kingston, ON, Canada; 2 Department of Community Health and Epidemiology, Queen's University, Kingston, ON, Canada; 3 Department of Laboratory Medicine, Saint John Regional Hospital, Horizon Health Network and Department of Pathology, Dalhousie University, Halifax, NS, Canada

## Abstract

**Background:**

The methylation of DNA is recognized as a key mechanism in the regulation of genomic stability and evidence for its role in the development of cancer is accumulating. LINE-1 methylation status represents a surrogate measure of genome-wide methylation.

**Findings:**

Using high resolution melt (HRM) curve analysis technology, we have established an in-tube assay that is linear (r > 0.9986) with a high amplification efficiency (90-105%), capable of discriminating between partcipant samples with small differences in methylation, and suitable for quantifying a wide range of LINE-1 methylation levels (0-100%)--including the biologically relevant range of 50-90% expected in human DNA. We have optimized this procedure to perform using 2 μg of starting DNA and 2 ng of bisulfite-converted DNA for each PCR reaction. Intra- and inter-assay coefficients of variation were 1.44% and 0.49%, respectively, supporting the high reproducibility and precision of this approach.

**Conclusions:**

In summary, this is a completely linear, quantitative HRM PCR method developed for the measurement of LINE-1 methylation. This cost-efficient, refined and reproducible assay can be performed using minimal amounts of starting DNA. These features make our assay suitable for high throughput analysis of multiple samples from large population-based studies.

## Background

Epigenetic refers to the study of heritable cellular changes that leave the DNA nucleotide sequence intact [1]. Increasingly it is recognized that epigenetic mechanisms play an important role in cancer etiology through alterations in gene activation, chromosomal stability, and genomic imprinting [2,3]. The methylation of DNA is recognized as a key epigenetic mechanism in the regulation of genomic stability and cell growth, and evidence for its role in the development of a wide variety of cancers is accumulating [4-10].

DNA methylation involves the transfer of a methyl group to cytosine in CpG (cytosine-phosphate-guanine) dinucleotide pairs. DNA methyltransferases are enzymes responsible for either creating or maintaining methylation patterns [11,12]. Two types of aberrant methylation patterns have been studied in relation to cancer etiology. Global hypomethylation, which refers to a genome-wide decrease in the number of cytosine bases that have been methylated to form 5-methyl-cytosine at CpG sites, is recognized as an early and consistent event in colorectal carcinogenesis [13-15]. Global hypomethylation is often accompanied by a second fundamental abnormality in DNA methylation, that is, gene-specific hypermethylation of CpG islands within the promoter regions of critical genes, which can lead to transcriptional silencing of tumor suppressor genes [16].

The majority of CpG nucleotides (about 80%) are found in repetitive nucleotide sequences, multiple copies of DNA elements that are normally methylated [17]. LINE-1 (long interspersed nucleotide element-1) sequences comprise approximately 20% of the genome and are the most widely studied repetitive sequences within the context of DNA hypomethylation and early colorectal cancer development [18,19]. Many studies have demonstrated that LINE-1 methylation status closely parallels overall global methylation levels, indicating that LINE-1 methylation levels represent a valid surrogate measure of genome-wide methylation [20-24].

Current evidence suggests that LINE-1 hypomethyation is not a passive change but contributes to neoplastic and other disease processes. While previously thought to be "junk" DNA, LINE-1 elements are now recognized as having played a key role in producing human genomic diversity. Through its retrotransposon activity, which acts on itself and other interspersed repetitive elements, LINE-1 elements have created an estimated 30% of the mass of the human genome [25]. Hypomethylation influences the activity of LINE-1 elements within the genome in a sequence- and position-dependent manner. There is also evidence that delayed or abnormal replication (i.e. independent repair of chromosome breaks) occurs more frequently if the DNA is hypomethylated [26]. Hypomethylation of LINE-1 sequences may be markers of regions of the genome at increased risk of error-prone DNA repair. Hypomethylation increases activity of the bidirectional LINE-1 promoter, and LINE-1 transcripts in *cis *can suppress the transcription of genes, including those involved in DNA repair [27,28]. Kaer et al. [29] found that active intronic LINE-1 sequences could cause transcriptional interference of genes through intron retention, forced exonization and cryptic polyadenylation.

All LINE-1 elements are not equally methylated (reviewed in [28]). Recently, researchers have focused on LINE-1 loci-specific methylation pattern analysis whereby individual LINE-1 CpG loci are classified into one of three classes of distinct methylation levels (hypomethylated, partially methylated and hypermethylated). Hypermethylation and partial methylation is characteristic of the majority of LINE-1 sequences in normal cells, while increased hypomethylation is observed in cancer. This repetitive loci-specific methylation measure has emerged as a promising potential clinical marker for the detection of cancer-related changes in DNA [28,30].

For the purposes of this research, our goal was to obtain a surrogate measure of global LINE-1 methylation based on a representative sub-set of CpG sites within the LINE-1 consensus promoter region that could be used as a determinant of risk in cancer prediction. An eventual application of this marker may be to identify people at higher risk for specific cancer types in order to target them for screening or intervention programs. In order to make use of a marker in this manner, it is necessary to evaluate the relationship between the marker and cancer risk in large population-based studies.

The objective of the current investigation was to design a rapid, reproducible and cost-effective method to estimate overall LINE-1 methylation that is suitable for a large population-based study with limited amounts of DNA available, using high resolution melt (HRM) curve analysis technology - a quantitative methylation-sensitive real-time fluorescence-based PCR method [31-33]. Methods used to quantify global DNA methylation that were developed prior to HRM are generally more expensive and labour-intensive, and require larger amounts of high quality DNA. To date, HRM-based methods have mainly been used to quantify gene-specific methylation [31-33]; however, one study used HRM to measure LINE-1 methylation [34]. In this report we utilized a Roche LightCycler 480 (LC480) and Gene Scanning Software, which was originally designed for Single Nucleotide Polymorphisms (SNP), to develop a high-throughput and precise assay to obtain an overall LINE-1 DNA methylation level across a subset of CpG sites within the LINE-1 repetitive sequence as a surrogate measure of global methylation.

## Materials and methods

### Participant sample collection

A blood sample was collected into a 10 mL EDTA tube from 13 healthy fasting volunteers. Following collection, blood was placed on ice, centrifuged within 30 min to isolate the buffy coat and then stored at -20°C until use. One additional volunteer provided blood to be used as the internal control sample for all HRM analyses. All procedures pertaining to the collection and processing of samples have been approved by the Queen's University Health Sciences Research Ethics Board and the Queen's University Biohazards Committee. Informed consent forms were successfully obtained from all participants in this report.

### Methylation standards

Human Methylated and Non-Methylated DNA standards were purchased from Zymo Research (Irvine, CA, USA), and used as received. Unless indicated, 2 μg of each standard DNA was bisulfite-converted as described below. To generate a range of methylated and unmethylated DNA standards, the two Zymo control DNA standards were mixed in 0, 10, 20, 30, 40, 50, 60, 70, 80, 90 and 100% methylated to unmethylated template ratios.

### DNA isolation

DNA was isolated from peripheral blood leukocytes (buffy coat) and purified using the 5-PRIME DNA isolation kit (Inter Medico, Markham, ON, Canada) according to instructions provided by the manufacturer. Purified DNA was stored at -20°C until use.

### Bisulfite conversion

For DNA standards and participant samples, purified DNA was quantified on a NanoDrop ND-2000 UV-Vis spectrometer (Thermo Scientific, Wilmington, DE, USA). DNA was bisulfite-converted (BSC) using an Epitect Bisulfite Conversion Kit (Qiagen, Valencia, CA, USA) which selectively deaminates cytosine, but not 5-methyl-cytosine, to uracil. Chemical modification of this nucleotide leads to a primary sequence change in the DNA that permits detection of unmethylated cytosines from 5-methyl-cytosine. To test the reliability of using the NanoDrop to quantify the amount of DNA after bisulfite conversion, further experiments were conducted to compare this spectral absorbance method with qPCR using a commercially available standard BSC DNA (D5015, Zymo Research, Irvine CA, USA) supplied at a concentration of 20 ng/μL to produce a qPCR standard curve. Comparisons were performed on three BSC DNA samples measured in triplicate (Methylated DNA Standard sample, Unmethylated DNA Standard sample, and internal control sample). No statistical difference was observed (*p *= 0.1814 using Paired *t*-test) when comparing these two methods of quantifying amounts of BSC DNA (See Additional file 1: Figure S-1 and Table S-1 for details). In addition, qPCR was used to verify that the concentrations of BSC DNA for the 100% and 0% methylated standards that were used to prepare the HRM standard curve were equal. The NanoDrop ND-2000 was used for all subsequent experiments to quantify BSC DNA. Column-purified BSC DNA was re-quantified on the NanoDrop ND-2000. All BSC DNA, unless stated otherwise, was diluted to 1 ng/μL for use in PCR.

### Influence of amount of starting DNA and BSC DNA

To evaluate the influence of the amount of starting DNA on LINE-1 methylation, DNA from the internal control blood was used at concentrations of 0.5, 1.0 and 2.0 μg. Three aliquots of each quantity of genomic DNA were tested and HRM analysis was done in triplicate on each of the three aliquots of each concentration. Similarly, the effect of different starting amounts of template BSC DNA on LINE-1 methylation levels was tested using 0.1-10 ng of BSC DNA template with one aliquot analyzed in triplicate for each quantity of starting BSC DNA.

### Details of HRM method and analysis of data

#### Primer Design

Primers were designed according to the recommendations of Wojdacz et al. [35-38] in order to minimize PCR bias. Briefly, primers were designed with the following conditions: i) primers must contain at least a single CpG site as far as possible from the 3' end of each primer, and ii) when possible, one or more natural thymidine nucleotides (T) originating from a non-CpG cytosine at the 3' end of each primer, was included. Inclusion of a CpG site in the 5' end of the primer permits experimental manipulation of the PCR bias towards the methylated DNA. The PCR bias can be modified by varying the annealing temperature during the amplification stage of the experiment. Inclusion of a natural thymidine increases the likelihood of amplifying only bisulfite-modified template DNA.

In the present study, primers were designed against the completely methylated sense strand sequence. The promoter region of the consensus LINE-1 sequence (GenBank #: X58075) was used to design primer sets with PRIMER DESIGNER 2.01 software (Scientific and Educational Software, Cary, NC, USA). This primer pair included one CpG dinucleotide in the 5' end of the forward primer and two CpG dinucleotide pairs at the 5' end of the reverse primer (Table 1). The 141 bp amplicon included a total of 8 CpG dinucleotides between the primers. The LINE-1 consensus promoter region, from which our 8 CpG sites of interest were selected, has been validated as representative of global DNA methylation status [22].

**Table 1:** Primer Sequence set of LINE-1

	Primer sequences (CpG sites in bold)	Annealing temperature (°C)	Amplicon size (bp)	Number of CpGs between primers
Primer Set	F: 5'-G**CG**AGGTATTGTTTTATTTGGGA-3' R: 5'-**CG**C**CG**TTTCTTAAACC-3'	54°C	141	8

#### HRM analysis

PCR amplification of the DNA was carried out using a Roche LightCycler 480 (Roche Applied Science, Laval, PQ, Canada) equipped with the Gene Scanning software (Version 1.5.0). PCR was performed in a 12 μL reaction volume and 2 ng of BSC DNA templates (2 μL) were added to each well which contained 1 × LightCycler 480 High Resolution Melting (HRM) Master Mix^® ^(Roche), 3.0 mM MgCl_2 _and 0.2 μM of each primer. The cycling protocol conditions included a single enzyme activation step of 10 min at 95°C followed by 40 cycles of the following steps: denaturation 95°C, 10 s, annealing 48°C, 10 s, and extension 72°C, 15 s. It is important to note that a touchdown PCR protocol as recommended by the LC480-HRM Master Mix was not adopted. Inclusion of a touchdown PCR protocol would have favoured the PCR bias towards the higher melting and thermodynamically more stable methylated DNA product.

The high resolution melting (HRM) step was performed after 40 cycles of amplification. The HRM analysis was initiated by denaturing all products at 95°C for 1 min, followed by annealing at 40°C for 1 min. Samples were quickly warmed to 55°C and then slowly warmed to 95°C at 0.1°C per second. Fluorescence data were collected at 25 acquisitions per second. The LC480-HRM Master Mix^® ^employed a saturating dye (ResolLight™, Roche) which facilitated the precise measurement of the melt curves of the amplicons. The Roche Gene Scanning software was employed for end product analysis. This algorithm allowed the raw melt curves to be normalized for fluorescence intensity, and a temperature shift was applied to align the normalized melt curves, which facilitated the analysis of samples with varying crossing point (Cp) values. A difference curve was then derived from the first derivative of the melt curves. Data for the difference melt curves were exported to Excel (Office 2007; Microsoft Corp., Redmond, WA, USA) for further analysis after the graphs were plotted and inverted vertically. Both peak-height and area-under-the-curve from the normalized, temperature-shifted, difference curves were used to generate a standard curve and determine the degree of methylation of each DNA sample.

#### Methylation analysis of participant samples

All participant DNA samples were analyzed on a 96-well plate which included a no-template control (NTC), a set of reference methylation standards, and an inter-assay internal control sample. Reference methylation standard curves and experimental samples were tested in triplicate. Standard curves were plotted with Prism (V4.03; GraphPad Software Inc., La Jolla, CA, USA), and percent methylation of each test sample was determined by interpolation of the data generated from the linear regression analysis of the standard curve from each plate.

### Intra- and inter-assay reproducibility of LINE-1 methylation

To assess reproducibility, blood samples from 13 participants were analyzed in triplicate on each of three plates. An identical internal control sample and a set of methylation standards were included on each plate. Objective criteria were established to determine when individual participant methylation values would be excluded from the analysis. For the HRM analysis, the first derivative of the melt curve was determined for each sample. Replicate samples were aligned and the peak-melt temperature for each sample within a set of triplicates was examined. A triplicate methylation value would be excluded from further analysis if the peak-melt temperature varied by more than 0.2°C from the other two remaining triplicate values. In addition, individual outliers (> 5% difference from each of the remaining triplicate values) would be removed and the average methylation value calculated using the remaining replicates.

The decision to re-run a plate was based on the performance of the internal control sample. The 5% difference rule described above was applied to the internal control triplicate values. If the difference between a specified pre-determined methylation cut-off level for the internal control (i.e. 90.5% methylation) and the average methylation value for the remaining internal control replicates for a specific plate was more than 5%, then the plate would be repeated.

Intra-assay reproducibility (e.g. within-sample reproducibility) was assessed with a one-way analysis of variance (ANOVA) [39,40]. The repeats of participants on separate plates were treated as separate samples. To assess inter-assay reproducibility (e.g. between-plate reproducibility) an average value was calculated for each sample on a plate. The coefficient of variation percentage (e.g. CV% = ratio of within-subject variance (Root mean square error) to the grand mean, multiplied by 100) were used to quantify intra- and inter-assay reproducibility.

## Results

### Assessment of methylation standards using HRM

**Figure 1:**
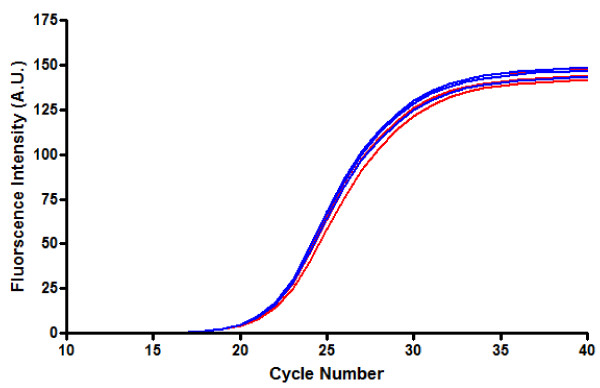
**Fluorescence curves (in triplicate) of fully methylated (red) and fully unmethylated (blue) BSC DNA standards**. It is important that both methylated and unmethylated DNA are amplified with similar efficiencies, thereby indicating no PCR bias for either template DNA.

The suitability of using methylation specific (MS)-PCR for measuring biologically significant levels of LINE-1 methylation was assessed by HRM PCR. Typically, primer pairs that produce reactions with Cp values of less than 30 cycles are suitable for subsequent HRM analysis. As illustrated in Figure 1, the Cp values as determined by the second derivative maximum of the fluorescence amplification curves are identical for both the methylated and unmethylated BSC DNA standards. This indicates that there is no PCR bias between the methylated and unmethylated DNA.

**Figure 2:**
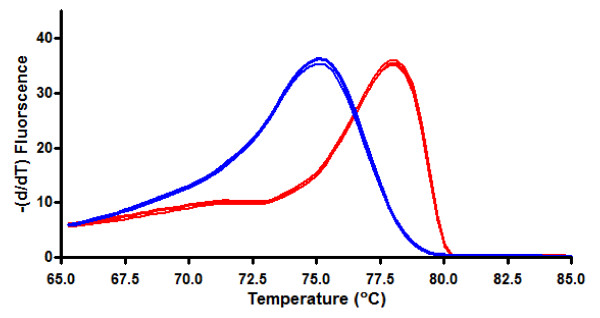
**Raw Melt curves (in triplicates) for 100% methylated (red) and 100% unmethylated (blue) BSC DNA standards**. Melt curve peaks differed by approximately 2°C and had characteristically different shapes indicating that the methylated and unmethylated DNA are different products.

A high resolution melt curve was generated from the amplified product using only the raw data. Figure 2 shows the raw first derivative of the HRM curve for two distinct products, the 0% and 100% methylated DNA standards. The melt curves generated using raw data displayed a Gaussian distribution, indicating that the amplicons consisted of a group of PCR products with similar melt characteristics for each of the methylated and unmethylated DNA standards. The 100% methylated BSC DNA had a melt peak at 77.8°C while the unmethylated BSC DNA melted at a lower temperature (75.3°C).

The Roche LC480 Gene Scanning software, originally developed for single nucleotide polymorphism (SNP) analysis, was utilized for end product analysis. The fluorescence melt curves were scaled and then normalized for fluorescence intensity. A temperature shift was then applied to align the normalized melt curves. The first derivative of the melt curves was calculated and a resultant difference curve plotted. In this study, a 0% methylated standard was used as the reference curve.

### Deriving the standard curve for determination of LINE-1methylation

**Figure 3:**
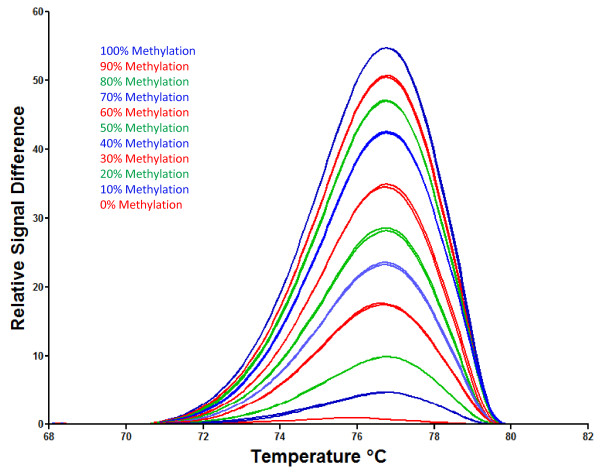
**Representative standard curves for the determination of methylated DNA**. Standard samples, from top to bottom, were 100, 90, 80, 70, 60, 50, 40, 30, 20, 10 and 0% methylated. Duplicate standard curves were plotted for each standard.

In order to accurately measure the percent methylation of DNA, a standard curve was established. Various amounts of BSC methylated and unmethylated DNA standards were mixed and assayed to produce a standard curve. HRM PCR was used to amplify BSC DNA standards. The resultant data were analyzed by the LC480 Gene Scanning software. The first derivatives of the difference plots were normalized and temperature-shifted, and the resultant difference plots for each standard were plotted in Figure 3. This graph shows that a range of 0%-100% methylated DNA can be resolved by this method. More importantly, the standards that represent biologically relevant methylation levels (50-90%) of LINE-1 methylation can also be resolved by this method.

### Quantification of LINE-1 methylation

**Figure 4:**
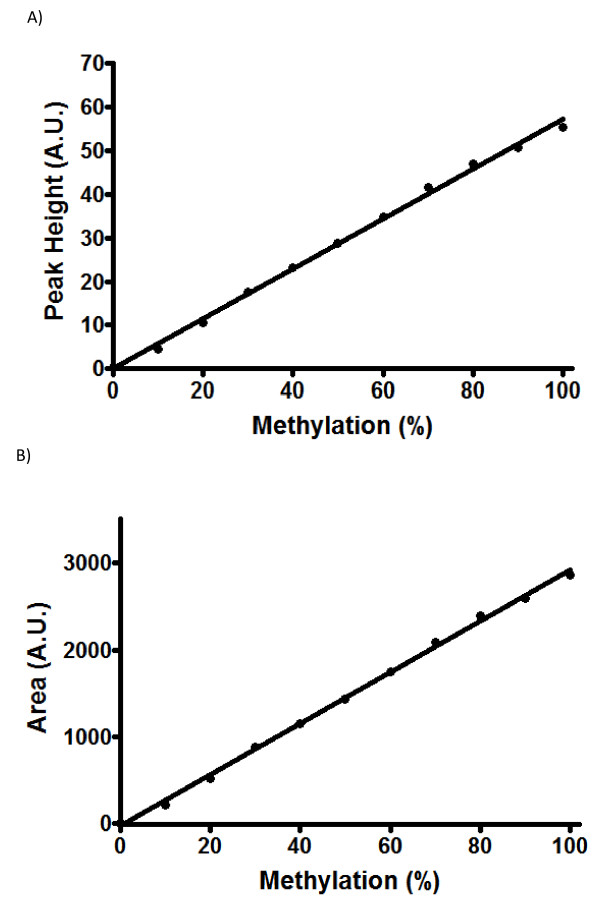
**A linear regression plot of the standard curves against percent methylation**.(A) A plot of peak-height versus percent methylation (r = 0.998). (B) A plot of area-under-the-curve versus percent methylation (r = 0.998). For this analysis, prepared standard samples with 0, 10, 20, 30, 40, 50, 60, 70, 80, 90 and 100% methylation were analyzed. All data points were plotted as mean of triplicate samples ± S.D. Due to minimal variation in triplicate measures, error bars were not visible in the plot.

Using the curves from Figure 3, the peak-height (Figure 4A) and the area-under-the-curve (Figure 4B) were used to generate two separate standard curves for quantification of percent methylation in order to determine which of the above methods was better to generate a standard curve for subsequent quantification. A linear regression analysis was used to plot a standard curve for subsequent analysis. The correlation coefficient, r = 0.9983, was determined from the plot of peak-height versus percent methylation, and the plot of area-under-the-curve versus percent methylation resulted in a plot with r = 0.9986, indicating a high precision across the full range of DNA methylation for both peak-height and area-under-the-curve. As can be seen from this analysis, both peak-height and area-under-the-curve plots can be used to quantify methylation. Moreover, both methods of analysis generate a linear fit which can be readily applied for accurate determination of LINE-1 methylation.

### HRM assessment of blood samples

#### Relationship between quantity of starting genomic DNA and LINE-1 methylation levels

**Figure 5:**
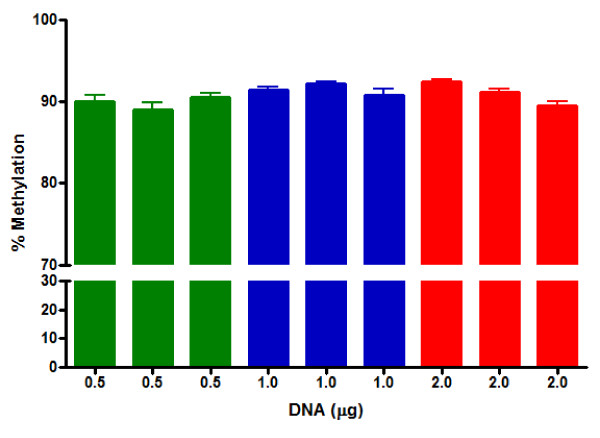
**Relationship between the starting amount of genomic DNA used for bisulfite conversion and percent methylation**. Column-purified BSC DNA was diluted to 1 ng/μL and used as a DNA template for HRM analysis for LINE-1 methylation. Values are expressed as mean percent ± S.D.

HRM analysis was used to measure the percent methylation of BSC DNA derived from varying amounts of starting DNA using the internal control blood sample. Three aliquots of each DNA starting amount were analyzed in triplicate. Figure 5 shows that regardless of the amount of starting DNA (0.5-2.0 μg) used for bisulfite conversion, the HRM analysis for LINE-1 methylation essentially produced similar results. Data were analyzed by one-way ANOVA with Tukey's *post hoc *test. There was no significant difference in methylation levels for each starting amount of DNA. Two μg of starting DNA were used for subsequent analyses using participant blood samples. The higher amount allowed a more precise quantification of DNA concentration using the Nanodrop Spectrometer.

#### Relationship between amount of BSC DNA template and LINE-1 methylation

**Figure 6:**
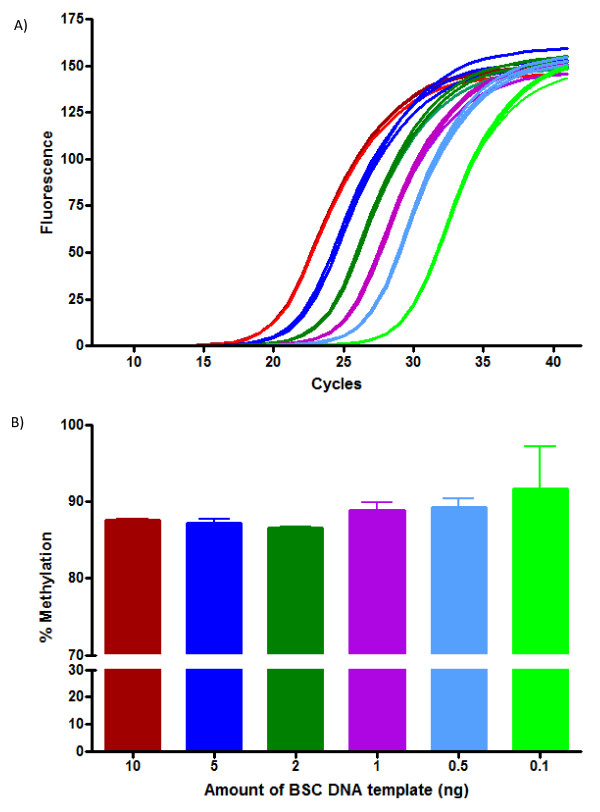
**Relationship between the amount of BSC DNA template from blood and LINE-1 methylation**. The amount of BSC DNA was varied (left to right, 10 (red), 5 (blue), 2.0 (green), 1 (purple), 0.5 (light blue) and 0.1 ng (light green)) and HRM was used to measure LINE-1 methylation. (A) Fluorescence curves for varying amounts of DNA. Each sample was analyzed in triplicate. Note that at 40 cycles, the 0.1 ng curve did not fully reach a plateau. (B) Determination of percent methylation as a function of amount of template BSC DNA. Values are expressed as mean percent ± S.D. Data were analyzed by one-way ANOVA with Tukey's *post hoc *test.

Figure 6 shows a titration curve with varying amounts of BSC DNA from a blood sample. One aliquot was analyzed in triplicate for each starting amount of BSC DNA. The Cp values were directly proportional to the amount of DNA template from 0.1 to 10 ng. A standard curve generated from this analysis had a slope of -3.858 and an efficiency of 1.89. Data were analyzed by one-way ANOVA with Tukey's *post hoc *test. No significant difference was determined between any of the groups, however the standard deviation of each sample was larger in the 0.1, 0.5 and 1.0 ng samples.

To further test the reliability of HRM for measuring methylation using lower amounts of BSC DNA, additional experiments were conducted comparing methylation levels for 13 participants using 0.5 ng versus 2.0 ng of starting BSC DNA. No statistical difference in methylation values was observed (*p *= 0.2673 using the Paired *t*-test) indicating that reliable methylation results can be obtained using this method with as little as 0.5 ng of starting BSC DNA.

In the current report, in order to reduce the standard deviation for a given sample, 2 ng of BSC DNA was chosen as the optimal amount for further experiments using methylation standards and participant samples. Similarly, individual aliquots of internal control DNA were prepared using 2 μg of starting DNA and 2 ng of BSC DNA for each subsequent HRM analysis.

### LINE-1 methylation and reproducibility in participant samples

Percent LINE-1 methylation in the 13 healthy participants, aged 42-64, ranged from 79.98% to 87.51% with a mean of 83.70%. The shape and pattern of the melt curves for participant samples was similar to the standard curves (See Additional file 2: Figures S-2 and S-3).

The evaluation of intra-assay and inter-assay reproducibility of this method is presented in Table 2. Each plate included a set of reference methylation standards, an internal control sample and participant samples. Each sample was done in triplicate. Objective criteria (described in Materials and Methods) were used to determine when to reject individual sample methylation values or entire plates. In this experiment, none of the triplicate values or plates was rejected.

**Table 2:** Reproducibility of LINE-1 methylation

	Samples	Range of Std Dev	Root Mean Square Error	Grand Mean	CV%
Intra-assay variation*	**39**	0.14-2.29	1.21	83.70%	1.44%

Inter-assay variation**	**13**	0.10-0.70	0.41	83.70%	0.49%

*Analysis included 3 replicates for each of the 13 participants on each of 3 plates (39 samples)

**Averages of 3 replicates for each of the 13 participants were compared between 3 plates (13 samples)

In the evaluation of reproducibility of replicates within a plate (within-subject or intra-assay variation) the blood samples from each of the 13 participants on each of the 3 plates were treated as separate samples. As a result, this analysis included 39 samples with 3 replicates for each sample. The coefficient of variation was 1.44% (Table 2).

To assess the inter-assay (between-plate) reproducibility, blood samples from each of the 13 participants were analyzed in triplicate on 3 separate plates and an average value was obtained for each sample on each plate. The inter-assay coefficient of variation (inter-assay variation) was 0.49%.

## Discussion

The analysis of repetitive elements in the human genome by PCR-based methods is a challenge as success requires identification of consensus primers and amplification conditions which can accommodate the sequence variation of the many copies of the repeat. The quantitative analysis of the methylation status of repetitive elements is technically more complex because of the need to bisulfite modify the DNA prior to amplification, which compounds the variability of the target sequences, along with the need for the assays to be as reproducible, precise and efficient as the equivalent analysis of single copy sequences.

We have established a refined, rapid and reproducible HRM PCR method that is suitable for quantifying global LINE-1 methylation in DNA isolated from peripheral blood leukocytes as a surrogate marker for measurement of global genomic methylation. Our method is linear, quantitative and highly repeatable. This relatively simple, single in-tube assay is capable of discriminating between participant samples with small differences in methylation values and quantifying a wide range of LINE-1 methylation levels (0-100%). The range of methylation values that we observed (approx. 80-88% with a mean of 84%) was consistent with blood leukocyte methylation values reported by Zhu et al. [41]. We have optimized the procedure so that it is possible to perform this assay using as little as 0.5 μg of starting amount of DNA per bisulfite conversion reaction and 0.5 ng of BSC DNA per PCR reaction while retaining an amplification precision of greater than 99% and efficiency between 90 and 105%. Both the intra-assay and inter-assay coefficients of variation were below 1.5%, supporting the high reproducibility and precision of this approach.

DNA methylation plays an essential role in maintaining cellular function, and changes in global methylation levels may contribute to the development of cancer. Several methods have been developed to quantify global DNA methylation [16,42-44] including enzymatic digestion followed by high performance liquid chromatography (HPLC) [45], traditionally considered to be the 'gold standard', and high performance capillary electrophoresis (HPCE) [46]. These techniques are generally expensive, labour-intensive and require fairly large amounts of starting DNA. Also, given that many population-based studies have large numbers of participant samples and often limited amounts of DNA, using these methods to measure methylation may be cost-prohibitive and technically challenging.

Methylation of the promoter region of LINE-1 repetitive elements has been investigated as a surrogate marker for global methylation levels using varied experimental approaches, including PCR-based methods such as Combined Bisulfite Restriction Analysis (COBRA), MethyLight and pyrosequencing [22,41,46-51]. These PCR-based methods use bisulfite-treated DNA in order to differentially modify methylated and non-methylated DNA. The COBRA assay uses amplification of bisulfite-modified DNA followed by restriction endonuclease digestion. A limitation of this method is the need for specific restriction enzyme recognition sequences within the target region. LINE-1 methylation levels quantified using MethyLight, a quantitative real-time Taqman-based assay, were highly correlated with genome-wide LINE-1 methylation as measured using HPLC [22]. Drawbacks to this method however are the need to use more costly Taqman dual-labeled hydrolysis probes and a reference assay for normalization [22]. Pyrosequencing has been used extensively for LINE-1 methylation analysis [41,50,51]. While pyrosequencing provides reliable counts of the methylation status at each CpG dinucleotide site within a small target region it does require specialized equipment. In addition, pyrosequencing is not able to provide information on the context of the methylation status of each site with respect to the others on the same DNA strand. Therefore subpopulations of sequences which exhibit very different methylation patterns cannot be distinguished.

High Resolution Melt analysis PCR (HRM PCR) is gaining acceptance as a robust method for measuring levels of methylation [52,53]. HRM utilizes the characteristics of the shape of the melting curve of BSC DNA to quantify methylation levels. By comparing a series of BSC dilution curves derived from varying mixtures of methylated and unmethylated DNA standards, the degree of methylation in a test sample can be determined [54]. HRM technology has primarily been used to quantify single site gene-specific DNA methylation [28,31,32]. Only one previous study has applied the HRM technology to measuring LINE-1 methylation in a variety of biological sample types [34]. Stanzer et al. [34] reported a high correlation between results obtained using HRM analysis compared to pyrosequencing and MethyLight analysis. However, the authors commented on achieving a modest linear dynamic range. Our study expands on the seminal work of Stanzer et al. [34] and provides additional experimental details useful for the adoption of this approach by other researchers.

There are limitations to HRM for analysis of LINE-1 methylation. For HRM studies of repetitive elements, the number of genomic targets is large. If a small proportion of the sequences present are extremely hypo- or hyper-methylated it may not be possible to distinguish them from the majority of LINE-1 targets [28,48]. In our study, the consistency of participant and standard HRM curves suggests that the level of methylation at the LINE-1 CpG sites of interest was uniform (See Additional file 2: Figures S-2 and S-3). A further potential limitation of our assay is the small number of CpG sites in the LINE-1 repetitive sequence that were analyzed. In order to maintain a high PCR efficiency, primers were designed to amplify the smallest fragment needed to include the 8 CpG dinucleotides in the target region. In support of this approach, the CpG sites within the LINE-1 promoter region that we selected have been validated by other methods as being representative of the total genomic methylation status [22].

Next generation sequencing (i.e., deep sequencing) is now permitting the study of entire genomes or targeted portions of genomes [55]. Through this technology, the location, sequence variation and methylation patterns of LINE-1 sequences and other repetitive elements throughout the genome are being established. While the detail on the heterogeneity of DNA methylation of LINE-1 and other sequences obtained with next generation sequencing technology will be unrivaled, at this time it is a costly approach for population-based studies which can require the assessment of thousands of samples. HRM technology provides a cost-effective and flexible means to survey the methylation status of single or multiple sites of interest, including repetitive elements, to identify potential markers of risk in studies of cancer prediction, or as a companion to other genomic, proteomic or metabolomic strategies to further understand the role of methylation in the normal functioning of the genome or in disease pathogenesis.

## Conclusion

In summary, we have established a refined, rapid and reproducible HRM-based method for the quantification of global LINE-1 repetitive element methylation that is able to address many of the technical, analytical and logistical challenges previously encountered. This assay covers the full range of methylation levels, and most importantly, methylation in the biologically relevant range of 50-90% in human DNA samples can be reliably quantified. The assay is linear, precise and efficient within parameters acceptable for any quantitative PCR-based assay using as little as 0.5 μg of starting DNA and 0.5 ng of BSC DNA for each PCR reaction. These features make our assay suitable for automation and rapid high-throughput analysis of multiple samples from large population-based studies. The use of this method in epigenetic research can be extended to the measurement of methylation levels of single copy sequences and other repetitive elements.

## Abbreviations

HRM: High resolution melt; LINE-1: Long interspersed nucleotide element-1; MS PCR: Methylation specific PCR; BSC DNA: Bisulfite-converted DNA; SNP: Single nucleotide polymorphism; MALDI-TOF MS: Matrix assisted laser desorption/ionization time of flight mass spectrometry; HPLC: High performance liquid chromatography; HPCE: High performance capillary electrophoresis; COBRA: Combined bisulfite restriction analysis.

## Competing interests

The authors declare that they have no competing interests.

## Authors' contributions

I. Concept and Design:MYT, JEA, WDK, SAMT, SCP, II. Acquisition of Data:MYT, JEA, NZ, SCP, III. Analysis and Interpretation of Data:MYT, JEA, NZ, WDK, SAMT, SCP, IV. Drafting and Revising the Manuscript:MYT, JEA, WDK, SAMT, SCP, V. Final Approval of the Manuscript:MYT, JEA, NZ, WDK, SAMT, SCP, VI. Funding of the Research Project:WDK, SCP. All authors read and approved the final manuscript.

## Supplementary Material

Additional file 1 **Figure S-1; Table S-1**. LINE-1 qPCR standard curve. Standard universal methylated bisulfite-converted DNA (20 ng/μL) (Cat. #: D5015, Zymo Research Corporation) was diluted with nuclease free water to produce a standard curve for the qPCR. DNA concentration was determined using the Absolute Quantification routine of the Roche LC-480 Software and our LINE-1 primer set. The latter is essential as we have demonstrated that this primer set does not have a PCR bias towards either methylated or unmethylated DNA. Cycling parameters for the qPCR were described in the Materials and Methods section and identical to the conditions of the HRM PCR; **Table S1: **Quantification of BSC DNA using UV spectrometry and qPCR. DNA concentration measured with the NanoDrop ND-2000 UV-VIS spectrometer were compared with values of DNA concentration measured by qPCR. Paired t-test analysis of the results from the two methods of quantification showed no significant difference between the concentration of DNA measured. These results demonstrated that the NanoDrop ND-2000 spectrometer is an accurate method for quantifying BSC DNA for HRM analysis. Click here for file

Additional file 2 **Figures S-2 and S-3**. Supplemental Figure S-2. Raw melt curves of 80, 90 and 100% methylation standard. This figure demonstrates the homogeneity of melt curves from 80, 90 and 100% of methylation standards, all in triplicates. Supplemental Figure S-3. Raw melt curves of blood sample from each of 13 subjects used in the current study. This figure demonstrates the homogeneity of melt curves in all subjects. Click here for file
