# Supplementary material for: A refined, rapid and reproducible high resolution melt (HRM)-based method suitable for quantification of global LINE-1 repetitive element methylation
Source: BMC Res Notes. 2011 Dec 28;4:565. doi: 10.1186/1756-0500-4-565 (PMC3284418; doi:10.1186/1756-0500-4-565)
Supplement: Additional file 1 — Figure S-1; Table S-1. LINE-1 qPCR standard curve. Standard universal methylated bisulfite-converted DNA (20 ng/μL) (Cat. #: D5015, Zymo Research Corporation) was diluted with nuclease free water to produce a standard curve for the qPCR. DNA concentration was determined using the Absolute Quantification routine of the Roche LC-480 Software and our LINE-1 primer set. The latter is essential as we have demonstrated that this primer set does not have a PCR bias towards either methylated or unmethylated DNA. Cycling parameters for the qPCR were described in the Materials and Methods section and identical to the conditions of the HRM PCR; Table S1: Quantification of BSC DNA using UV spectrometry and qPCR. DNA concentration measured with the NanoDrop ND-2000 UV-VIS spectrometer were compared with values of DNA concentration measured by qPCR. Paired t-test analysis of the results from the two methods of quantification showed no significant difference between the concentration of DNA measured. These results demonstrated that the NanoDrop ND-2000 spectrometer is an accurate method for quantifying BSC DNA for HRM analysis. [file 1756-0500-4-565-S1.PDF]

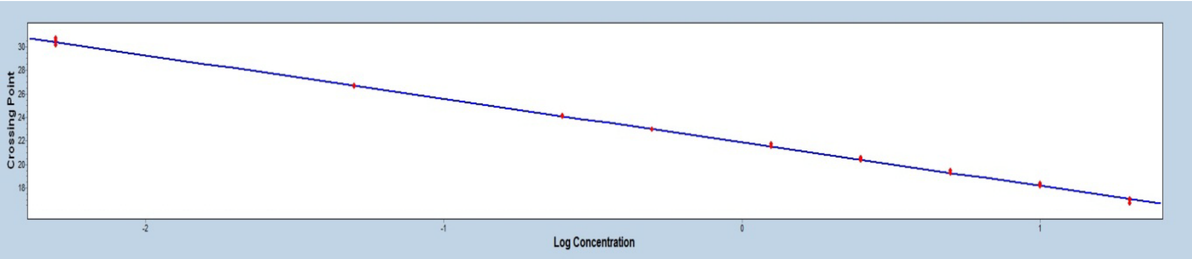

Figure S-1. LINE-1 qPCR standard curve. Standard universal methylated bisulfite-converted DNA (20 ng/µL) (Cat. #: D5015, Zymo Research Corporation) was diluted with nuclease free water to produce a standard curve for the qPCR. DNA concentration was determined using the Absolute Quantification routine of the Roche LC-480 Software and our LINE-1 primer set. The latter is essential as we have demonstrated that this primer set does not have a PCR bias towards either methylated or unmethylated DNA. Cycling parameters for the qPCR were described in the Materials and Methods section and identical to the conditions of the HRM PCR.

Table S-1. Quantification of BSC DNA using UV spectrometry and qPCR. DNA concentration measured with the NanoDrop ND-2000 UV-VIS spectrometer were compared with values of DNA concentration measured by qPCR. Paired t-test analysis of the results from the two methods of quantification showed no significant difference between the concentration of DNA measured. These results demonstrated that the NanoDrop ND-2000 spectrometer is an accurate method for quantifying BSC DNA for HRM analysis.

Sample	UV absorbance (ng/µL ± S.D.)	qPCR (ng/µL ± S.D.)
BSC Methylated DNA Standard	19.14 ± 0.73	19.07 ± 0.34
BSC Unmethylated DNA Standard	14.77 ± 0.51	14.17 ± 0.20
BSC Blood DNA	12.40 ± 0.79	11.42 ± 0.10
