# Supplementary material for: A refined, rapid and reproducible high resolution melt (HRM)-based method suitable for quantification of global LINE-1 repetitive element methylation
Source: BMC Res Notes. 2011 Dec 28;4:565. doi: 10.1186/1756-0500-4-565 (PMC3284418; doi:10.1186/1756-0500-4-565)
Supplement: Additional file 2 — Figures S-2 and S-3. Supplemental Figure S-2. Raw melt curves of 80, 90 and 100% methylation standard. This figure demonstrates the homogeneity of melt curves from 80, 90 and 100% of methylation standards, all in triplicates. Supplemental Figure S-3. Raw melt curves of blood sample from each of 13 subjects used in the current study. This figure demonstrates the homogeneity of melt curves in all subjects. [file 1756-0500-4-565-S2.PDF]

Raw Melt curve of 100% methylation Standard

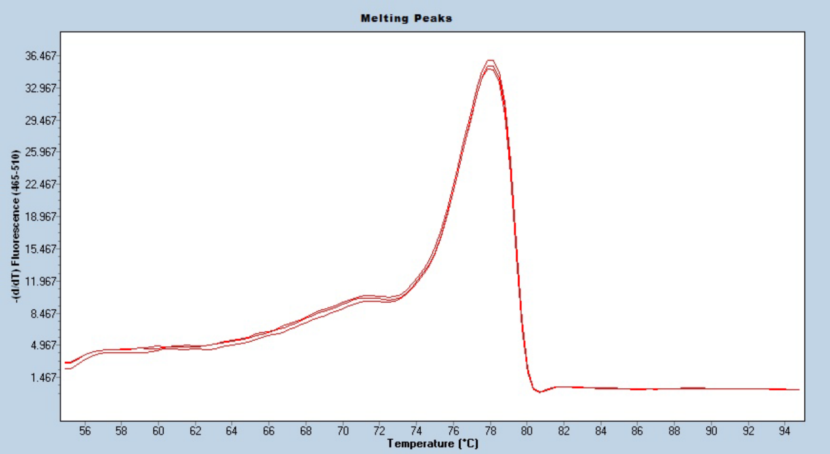

Raw Melt curve of 90% methylation Standard

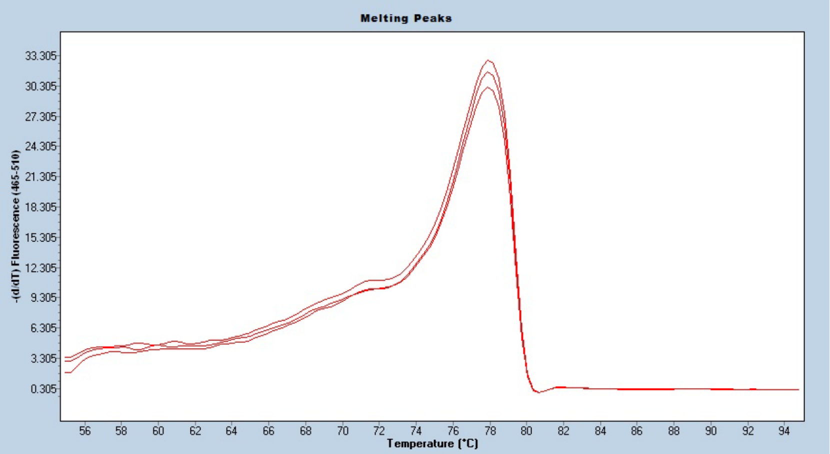

Raw Melt curve of 80% methylation Standard

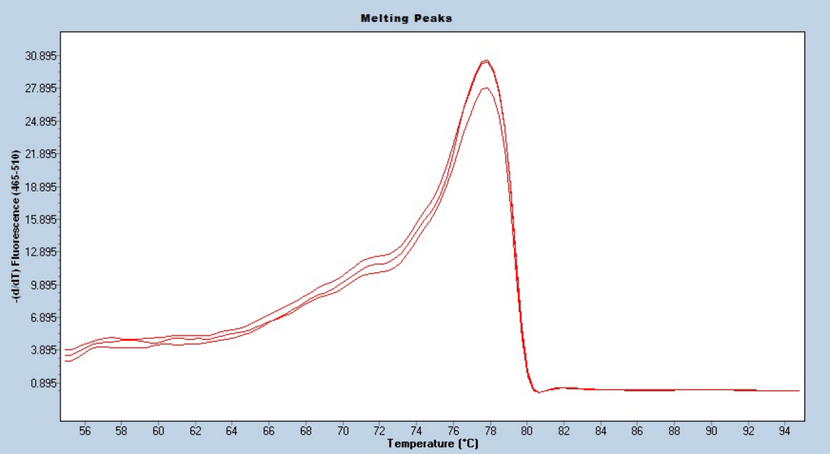

Gene Scanning Difference Melt Curves 100%, 90%, 80%

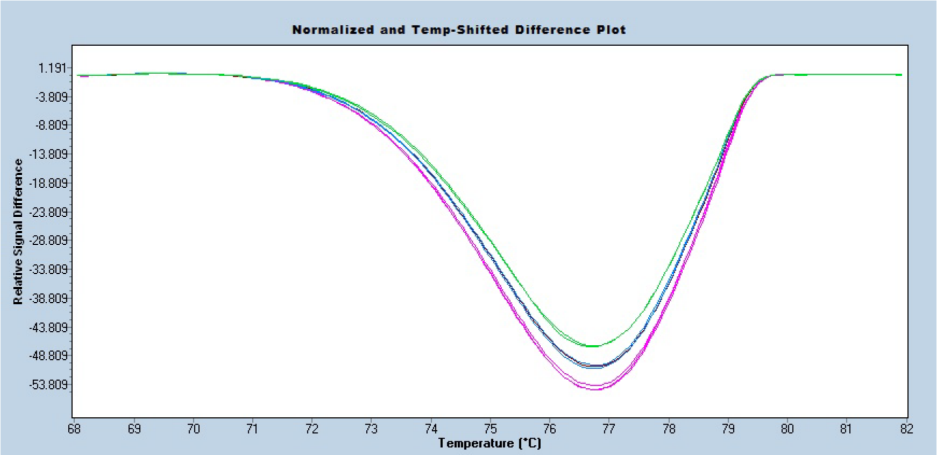

Supplemental Figure S-2. Raw melt curves of 80, 90 and 100% methylation standard. This figure demonstrates the homogeneity of melt curves from 80, 90 and 100% of methylation standards, all in triplicates.

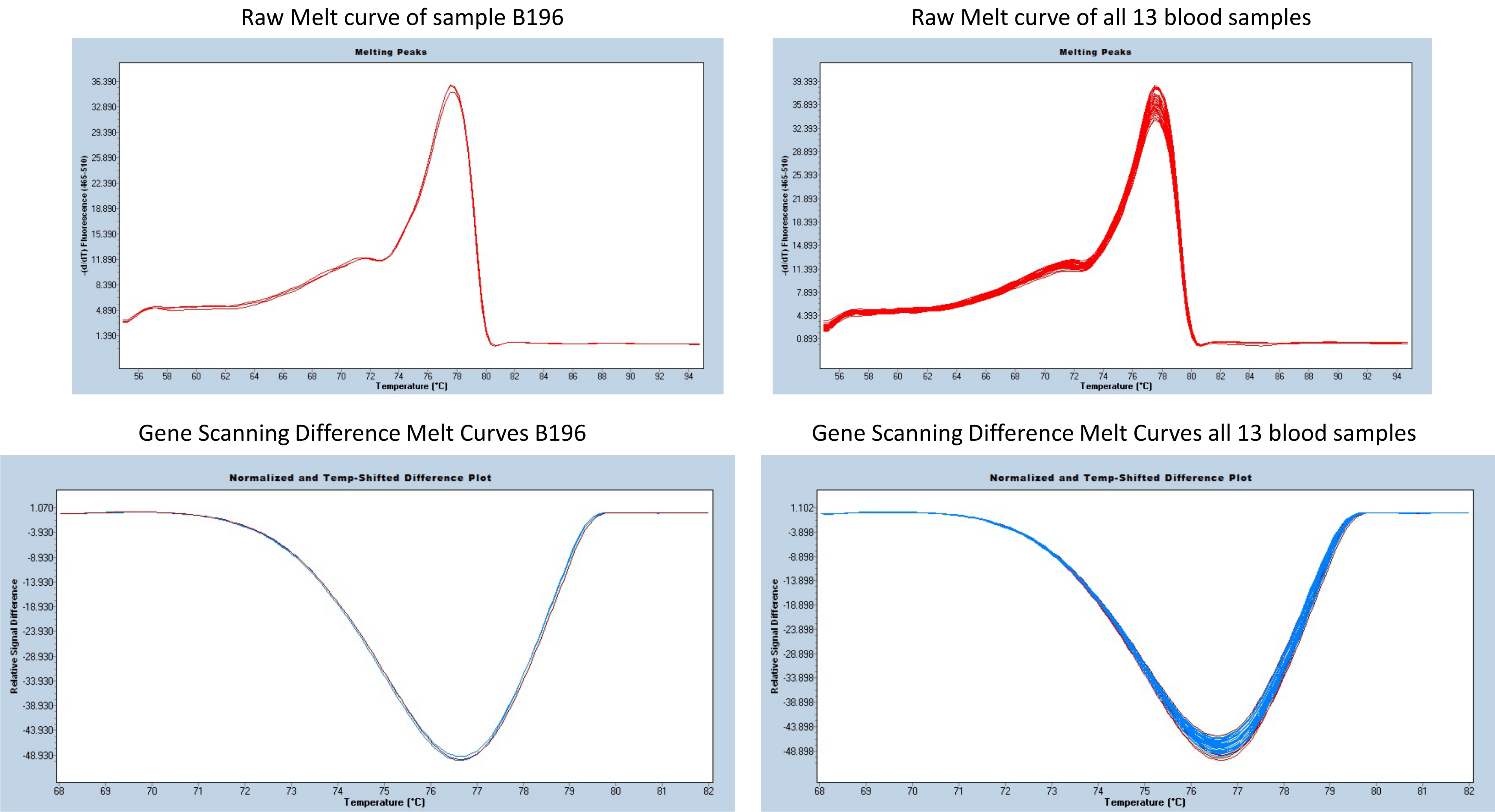

Supplemental Figure S-3. Raw melt curves of blood sample from each of 13 subjects used in the current study. This figure demonstrates the homogeneity of melt curves in all subjects.
